# A study of patients’ quality of life more than 5 years after trauma: a prospective follow-up

**DOI:** 10.1186/s12955-020-01652-1

**Published:** 2021-01-08

**Authors:** Fanny Vardon-Bounes, Romain Gracia, Timothée Abaziou, Laure Crognier, Thierry Seguin, François Labaste, Thomas Geeraerts, Bernard Georges, Jean-Marie Conil, Vincent Minville

**Affiliations:** 1 Anesthesiology and Critical Care Unit, Toulouse University Hospital, 1 av du Pr Jean Poulhès, 31059 Toulouse, France; 2 Inserm U1048, I2MC, Université Paul Sabatier, 31024 Toulouse Cedex 03, France

**Keywords:** Quality of life, Trauma, NHP, MOS-SF36, Disability, Rehabilitation

## Abstract

**Background:**

The long-term fate of severely injured patients in terms of their quality of life is not well known. Our aim was to assess the quality of life of patients who have suffered moderate to severe trauma and to identify primary factors of long-term quality of life impairment.

**Methods:**

A prospective monocentric study conducted on a number of patients who were victims of moderate to severe injuries during the year 2012. Patients were selected based on an Injury Severity Score (ISS) more than or equal to 9. Quality of life was assessed by the MOS SF-36 and NHP scores as a primary evaluation criterion. The secondary evaluation criteria were the determination of the socio-economic impact on quality of life and the identification of factors associated with disability.

**Results:**

Two hundred and eight patients were contacted by e-mail or telephone. Fifty-five patients participated in this study (with a participation level of 26.4%), including 78.2% men, with a median age of 46. Significant alterations in quality of life were observed with the NHP and MOS SF-36 scale, including physical and psychological components. This resulted in a major socio-economic impact as 26% of the patients could not resume their professional activities (n = 10), 20% required retraining in other lines of work, and 36.4% had a disability status. The study showed that scores ≤ 85 on the physical functioning variable of the MOS SF 36 scale was associated with disability.

**Conclusion:**

More than five years after a moderate to severe injury, patients’ quality of life was significantly impacted, resulting in significant socio-economic consequences. Disability secondary to major trauma seems to be associated with a score ≤ 85 on the physical functioning dimension of the MOS SF-36 scale. This study raises the question of whether or not early rehabilitation programs should be implemented in order to limit the long-term impact of major trauma.

## Background

Trauma is a frequent reason for admission to intensive care units (ICU) [1]. A recent study revealed a 30-day mortality of 5.9% in a retrospective French cohort of 144,058 trauma patients in which age and injury severity were the stronger predictors for mortality [2]. In the United States, severe trauma is the cause of 2 million hospitalizations and results in over 150,000 deaths. There is a major socio-economic impact, with medical fees for severely injured patients resulting in an estimated annual cost of 16 million dollars, while costs related to secondary disabilities from these traumas are estimated at 150 billion dollars annually [3].

Numerous studies conducted in patients with Acute Respiratory Distress Syndrome (ARDS) and/or sepsis [4–8], have helped to highlight the development of varying but often very frequent cases of functional and neuropsychological disorders after hospitalization in the ICU. The British health community defines this disorder as “PICS” for “Post Intensive Care Syndrome”. In an extensive research review including 19 articles, Wolters et al. [9] highlight the fact that 4 to 62% of patients present with cognitive impairments after care in the ICU.

However, in Europe few studies have shown interest in the outcome of patients who have suffered moderate to severe traumas [10].

Braithwaite et al. found that half of the patients who suffered from a major trauma developed moderate to severe disabilities [11]. Sluys et al. found significant physical and psychological impairments in respectively 68% and 41% of the patients [12]. The Von Rüden et al. study showed that 85% of the patients had an impaired capacity to work, and 62% lived with chronic pain. In addition to these results, 48% of the patients showed signs of clinical depression and 41% were victims of post-traumatic stress after experiencing trauma [10].

To our knowledge, no studies concerning the long-term outcome of patients with moderate to severe trauma have been conducted in France.

The main objective of our study was to assess the long-term outcome and quality of life in moderate to severe trauma patients managed in a Level 1 trauma center, more than five years after their injury. Secondly, we investigated the predictive factors for long-term alterations in their quality of life, as well as the socio-economic repercussions.

## Methods

### Study population

The study was conducted in the Level 1 trauma center at Toulouse University Hospital in 2017.

All patients aged > 16, admitted in Intensive Care Unit, who have been discharged alive after a moderate or severe trauma five years earlier were screened in the study. An ISS (Injury Severity Score) more than or equal to 9 qualified the trauma as moderate to severe. The exclusion criteria were patients dead at day 30, minors, and beneficiaries of judicial forms of protection.

The Toulouse University Hospital ethics and research committee approved this study (project number 08-0916). Informed consent was obtained through forms from all the participants in the study.

### Proceedings

All the medical and administrative data gathered on the potentially eligible patients were analyzed retrospectively.

All patients known to have survived major trauma were then contacted by mail, and then by phone.

Initially, all necessary documents were sent by mail, including various quality of life surveys, a written information form, an informed consent form and a stamped envelope for the main investigator, in order to collect all duly completed informed consent forms and surveys. In the second stage, once the initial documents were mailed to patients, a telephone call was made to gather additional information, and if the patients agreed, a complete interview was conducted to allow them to answer questions on the phone.

### Selection criteria

The primary endpoint of the study was the assessment of quality of life 5 years after the trauma. Therefore, two standardized scientifically approved scales were used: The Medical Outcome Study Short Form-36 (MOS- SF-36) and the Nottingham Health Profile (NHP). MOS SF-36 is a multidimensional, generic scale that assesses health status independently of causal pathology, sex, age, and treatment. This survey, made up of 36 different fields of inquiry, evaluates eight dimensions of health: physical functioning, limitations due to physical conditions, physical pain, mental health limitations due to mental status, social functioning, vitality and general health. An average physical score and an average emotional score can be calculated from these 8 dimensions according to an existing algorithm. The Nottingham Health Profile is a scale that was published in Britain at the end of the 1970s. The French version was edited and validated by Bucquet et al. [13] in 1990. Like the MOS SF-36, age and sex do not influence the value of the score. This scale is comprised of 38 items that assess 6 dimensions: mobility, social isolation, pain, emotional reactions, energy and sleeping habits. Scores rank from 0 to 100%.

In addition, another survey developed specifically for this study was provided to patients to subjectively describe their rehabilitation, the impact of their trauma on their professional lives, leisure, and disability status according to criteria from the French Health Insurance Agency. Factors associated with disability were the secondary endpoints.

### Acquired data

Data were retrieved from the Orbis® software (Agfa Healthcare, Bordeaux, France) which gathers all hospitalized patients’ medical and administrative information at the University Hospital Center.

The following data were collected:

- Demographic data: age, gender, nature of the initial injury, severity scores (SAPS II (New Simplified Acute Physiology Score II) and ISS (Injury Severity Score)), data relating to treatment plans followed (transfusion, surgical care, embolization, mechanical ventilation), the main biological parameters and the patient outcome (length of stay in intensive care units and length of hospitalization).
- Previous habits: professional career, driving, regular leisure activities.
- Follow-up information after moderate to severe trauma: number of repeat surgical procedures, number of specialized consultations, evaluation of rehabilitation (length of care, quality of rehabilitation, patient’s wish to be under the care of a trauma specialist such as an anesthesiologist).
- Aspects of post-trauma life: resumption of professional activity (work adjustments, partial or full-time job, necessity of professional retraining), the resumption of driving and leisure activities.
- Medical and economic factors: category of invalidity, receipt of disability pension, the average disability payment, employment of a care provider.

### Statistical analysis

- Characteristics of patients were described in terms of averages, minimum, maximum and median, first and third (25-75P) quartile or percentages according to the type of variables.
- For the MOS SF-36, the percentage was set at 100 for the maximal score (e.g. favorable) and 0 for the minimal score (e.g. unfavorable) and vice versa for the NHP score (minimal score 0/100 e.g. favorable, maximal score 100/100 e.g. unfavorable).
- The percentage of patients presenting with a severely altered quality of life was noted. For every dimension of the MOS SF-36, the minimal threshold that defined a severely altered quality of life was calculated from data observed in the French general population (source: INSEE (French Institute of Statistics and Economic Studies) study of health and medical care conducted in 2002–2003). The calculation of statistical significance was not possible due to the difference in the number of patients between the groups and the comparison is given for information purposes only.

The ROC (Receiver Operating Characteristics) curves and their areas under the curve were used to identify the discriminating value for the occurrence of disability. The choice of the most discriminating thresholds was achieved according to Youden’s index. After this univariate analysis that allows the individualization of covariates (continuous and nominal) associated with the occurrence of a disability, a multidimensional analysis facilitated the evaluation of different covariates and the disability variable by Risk-Ratio measurement. Results with an alpha *p* < 0.05 were considered statistically significant. Statistical analyses were performed using MedCalc® software (Ostend, Belgium).

## Results

**Fig. 1:**
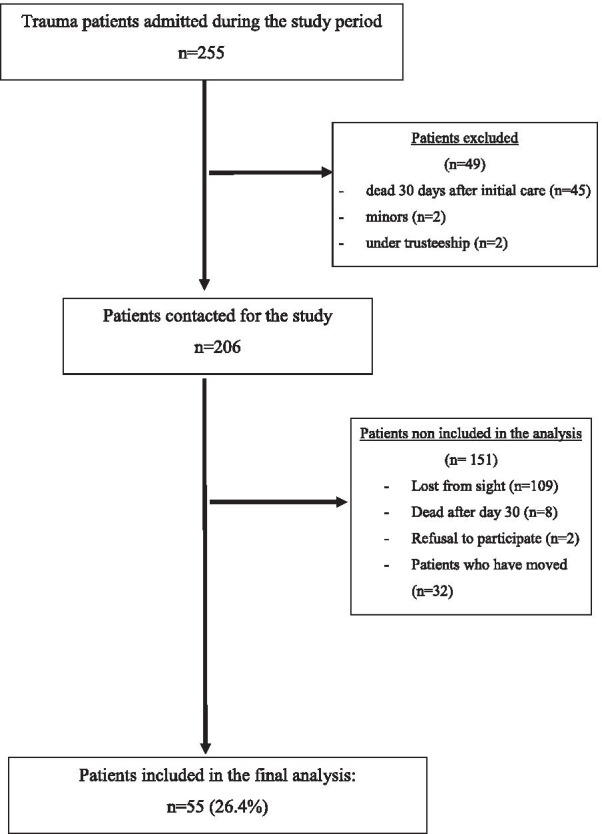
Flowchart of the study

Of the 255 patients initially screened in the study, 208 were contacted (Flow chart in Fig. 1). Finally, 55 patients responded to the questionnaire and were included in the final analysis. Demographics and surgical data are shown in Table 1. There was no statistical difference between the responsive and non-responsive population.

**Table 1:** Summary of clinical and biological data of the population studied

	Non-responsive population (n = 153)	Participating population (n = 55)	*p* value
*Epidemiological data*			
Age (years)	36 [16–88]	46 [17–76]	0.087
SAPS II	27 [6–65]	26 [7–66]	0.93
ISS	18 [9–41]	18 [9–41]	0.42
Gender ratio (Male/female)	114 (74.5%)/39 (25.5%)	43 (78.2%)/12 (21.8%)	0.59
*Biological data*			
pH	7.5 [7.02–7.99]	7.35 [7.19–7.5]	0.36
Serum lactate (mmol/l)	1.9 [0.55–12.9]	1.8 [0.4–9.85]	0.99
Hemoglobin (g/dl)	12.1 [4.5–16.89]	11.6 [7.5–16.8]	0.81
*Injury data (n, %)*			
Thorax	111 (72.5%)	38 (69.1%)	0.63
Skull	71 (46.4%)	31 (56.4%)	0.21
Limbs	78 (51%)	29 (52.7%)	0.83
Abdomen	46 (30.1%)	22 (40%)	0.18
Spine	43 (28.1%)	17 (30.9%)	0.70
Pelvis	41 (26.8%)	15 (27.3%)	0.95
Face	41 (26.8%)	12 (21.8%)	0.47
*Therapeutics*			
Transfusion	93 (61.2%)	36 (66.7%)	0.48
Emergency surgery	70 (47.3%)	29 (52.7%)	0.49
Length of mechanical ventilation (days)	1 [0–52]	1 [0–43]	0.33
Length of hospitalization in the ICU (days)	12 [1–93]	13 [3–63]	0.96
Length of overall hospitalization (days)	25 [2–123]	25 [3–108]	0.57

Results expressed in median and extreme values as well as numeric values and averages (%). SAPS II: Simplified Acute Physiology Score II, ISS: Injury Severity Score

### Medical follow-up and rehabilitation data

Following hospital care, 40 patients (74.1%) benefited from rehabilitation and 25 patients (46.3%) admitted regretting not having been followed by a trauma specialist such as an anesthesiologist and intensive care provider.

Concerning medical follow-up, the median number of repeat surgical procedures and consultations were respectively 2 [0–9] and 6 [0–31].

### Social and economic data

Twenty-six percent of the patients (n = 10) did not return to work after their accident. Resumption of full-time activity was permitted in 68% of the cases. Almost one out of five patients had to be re-trained in a different field of work, and 16.7% (n = 6) had to benefit from an adaptation of their workstation.

Similar to the impact on professional aspects of life, 39.5% of the patients (n = 17) reported having resumed their leisure activities on a regular basis (versus 70% prior to the accident). Driving was resumed in 94.5% of the cases (n = 52).

Finally, 36.4% (n = 20) of the responders were assumed to have a disability status according to criteria by the French Health Insurance Agency. The median rate of disability was 25%.

### Quality of life

The assessment of quality of life data is shown in Table 2 and was explored by the 2 scales mentioned above.

**Table 2:** Quality of life of the trauma group

	Average ± SD	Patients with minimal score/item (favorable)	Patients with maximal score/item (unfavorable)
*NHP*			
Pain	31.3 ± 36.8	22 (40.0%)	7 (12.7%)
Energy	40.8 ± 41.6	21 (38.9%)	15 (27.8%)
Social isolation	16.5 ± 30.9	38 (69.1%)	0 (0%)
Mobility	19.6 ± 25.4	24 (43.6%)	1 (1.8%)
Sleep	20.3 ± 28.8	27 (49.1%)	1 (1.8%)
Emotional reaction	26.1 ± 34.8	26 (47.3%)	3 (5.5%)
*SF 36*			
Physical pain	54.5 ± 35	13 (23.6%)	3 (5.5%)
Emotional well-being	43.5 ± 13.6	0 (0%)	0 (0%)
General health	55.6 ± 20.9	0 (0%)	0 (0%)
Physical functioning	65.5 ± 33.7	10 (18.2%)	4 (7.3%)
Social functioning	58.8 ± 45.3	10 (18.2%)	3 (5.5%)
Limitations due to emotional problems	59.5 ± 44.2	28 (50.9%)	17 (30.9%)
Limitations due to physical health	45.9 ± 19.4	26 (47.3%)	16 (29.1%)
Energy	58.8 ± 26.2	0 (0%)	0 (0%)
Average physical score	52.7 ± 20.7	0 (0%)	0 (0%)
Average emotional score	54.5 ± 35	0 (0%)	0 (0%)

Results expressed as mean and standard deviation. Number of patients with minimal and maximal scores is expressed in numeric values and percentages

After evaluation with the Nottingham Health Profile scale, the dimensions associated with significant impaired quality of life, therefore those most affected by trauma, were “energy” with 27.8% (n = 15) of the population admitting loss of energy and “pain” (12.7% of patients (n = 7) admitting major pain), respectively average values of 40.8 ± 41.6 and 31.3 ± 36.8 (0/100 corresponding to the best score and 100/100 corresponding to the worst score).

The least altered dimensions were “social isolation” (69.1% with minimal score stating that patients did not feel isolated), “sleep” (49.1% of minimal score) and “emotional reaction” (47.3% of maximal score) (Table 2).

With regard to the MOS SF-36 scale, the most unfavorable scores were related to the items “limitations due to emotional problem” and “limitations due to physical health” with respectively 30.9% and 29.1% of patients with minimal score.

An elevated percentage of maximal scores was observed in the “physical pain” (23.6%), “physical functioning” (18.2%) and “social functioning” (18.2%) dimensions with the following average scores 54.5 ± 35, 65.5 ± 33.7 and 58.8 ± 45.3 (0/100 corresponding to the worst score and 100/100 corresponding to the best score).

As an indication, we compared quality of life of the studied population to a reference group from an INSEE survey. Comparison is shown in Table 3. The calculation of statistical significance was not possible due to the difference in the number of patients between the groups and the comparison is given for information purposes only.

**Table 3:** Comparison between the trauma group and a reference group of mean (SD) SF-36 scores

SF-36 items	Trauma group N = 55	Reference group* N = 20,754	Difference between the reference and trauma patients
Physical pain	54.5 ± 35	73 ± 24.6	− 18.5
Emotional well-being	43.5 ± 13.6	66.7 ± 17.7	− 23.2
General health	55.6 ± 20.9	67.8 ± 18.9	− 12.2
Physical functioning	65.5 ± 33.7	85.3 ± 22.3	− 19.8
Social functioning	58.8 ± 45.3	80.9 ± 21.2	− 22.1
Limitations due to emotional problems	59.5 ± 44.2	82 ± 32.9	− 22.5
Limitations due to physical health	45.9 ± 19.4	82.2 ± 32.2	− 36.3
Energy	58.8 ± 26.2	57.4 ± 18	1.4
Average physical score	52.7 ± 20.7	50.3 ± 9.1	2.4
Average mental score	54.5 ± 35	47.2 ± 9.7	7.3

Results are expressed in averages and standard deviations for data from the INSEE study* and in numerical values with regards to the variation in averages in quality of life in both populations. The calculation of statistical significance is not possible due to the difference in the number of patients between the groups and the comparison is given for information purposes only

The results could suggest that there are alterations in all quality of life dimensions in patients who had suffered major trauma except for three: “energy”, “average physical score” and “average mental score”.

Physical dimensions could be the ones most affected by moderate to severe trauma.

Psychological dimensions, notably “emotional well-being” (− 23.2), “social functioning” (− 22.1) and “limitations due to emotional problems” (− 22.5) reveal lower scores than those of the general population.

This comparative analysis suggests that both the physical and psychological impact of moderate to severe trauma.

### Factors associated with disability

This study revealed an absence of statistically significant correlations between injury, location of initial injury and quality of life scores.

A comparative analysis was done on the clinical data and the quality of life scores for patients with a disability status and those who were considered able-bodied (Table 4).

**Table 4:** Comparison of the clinical data and quality of life scores of the able-bodied population and the population with a disability

	Able-bodied (n = 35)	Disabled (n = 20)	*p*
	Median (25–75 P)	Median (25–75 P)	
*Clinical data and length of stay*			
Age at occurrence of trauma (years)	48 (28.5–59)	44.5 (36–52)	0.75
Men/ Women n (%)	30 (85.7%)/5 (14.3%)	13 (65%)/7 (35%)	0.09
SAPS II	25 (18–37)	29 (19–40.5)	0.37
ISS	18 (13–25)	22 (13–26)	0.40
Emergency surgery	51.4%	55%	0.99
Length of time (days)			
ICU	11 (5–22.5)	14 (9.5–27.5)	0.28
Mechanical ventilation	0 (0–10)	4 (0–15)	0.29
In the hospital	20 (10.2–40.5)	28.5 (21.5–36.5)	0.09
Rehabilitation (months)	3 (2.25–6.5)	8 (4.75–29.2)	0.0113*
Resumption of professional activity	58.8%	42.1%	0.27
Employment of a caregiver	7(20%)	11 (55%)	0.015*
MOS SF-36			
Physical pain	70.5 (28.1–100)	25 (12.5–56)	0.0029*
Emotional well-being	45 (31.25–55)	42.5 (35–52.5)	0.94
General health	53 (44.7–75.7)	48.9 (39–71.7)	0.37
Physical functioning	90 (55–100)	50 (27.5–72.5)	0.0008*
Social functioning	87.5 (44.6–100)	41.5 (29–70.5)	0.0011*
Limitations due to emotional problems	100 (8.3–100)	33.3 (0–100)	0.05
Limitations due to physical health	100 (31.3–100)	25 (0–87.5)	0.0095*
Energy	43.7 (32.8–60.9)	43.75 (25–53.1)	0.29
Average physical score	73.7 (53–85.6)	43 (25–61.1)	0.0015*
Average emotional score	65.6 (42–72.9)	44.3 (25–60.7)	0.0124*
NHP			
Pain	0 (0–27.8)	45.9 (10.4–86.4)	0.0037*
Energy	0 (0–39)	100 (30.5–100)	< 0.0001*
Social isolation	0 (0–12.3)	0 (0–59.3)	0.16
Mobility	0 (0–11.5)	32,6 (11.5–52.7)	0.0001*
Sleep	0 (0–16.5)	13.95 (0–47.5)	0.42
Emotional reactions	0 (0–34.7)	35.4 (4.4–45.9)	0.0201*

Results expressed in medians and 1st and 3rd percentile as well as numerical values and averages (%) *p < 0.05

According to the statistical data, disabled patients required longer rehabilitation and needed personal assistance more often.

The NHP score corroborated results obtained with the MOS SF-36 score since the “pain” and “mobility” dimensions statistically decreased in the disabled group. Results also indicated significant decreases in the “energy” and “emotional reactions” dimensions.

Therefore, the results highlight significant discrepancies between the able-bodied population and the disabled population when comparisons were made between the MOS SF-36 scale and the NHP scale. Some dimensions (physical and emotional) were statistically lower in the disabled group. Therefore, we can hypothesize that these dimensions are predictive factors of disability and quality of life impairment after moderate to severe trauma.

Concerning the MOS SF-36 score, disabled patients had significant decreases for all physical dimensions (“pain”, “physical functioning”, “limitations due to physical health” and “average physical score”) compared to the group of able-bodied patients. Nevertheless, it was noted that the MOS SF-36 scale demonstrated a statistically significant difference between two items of the emotional dimension: “social functioning” and “average emotional score”.

**Fig. 2:**
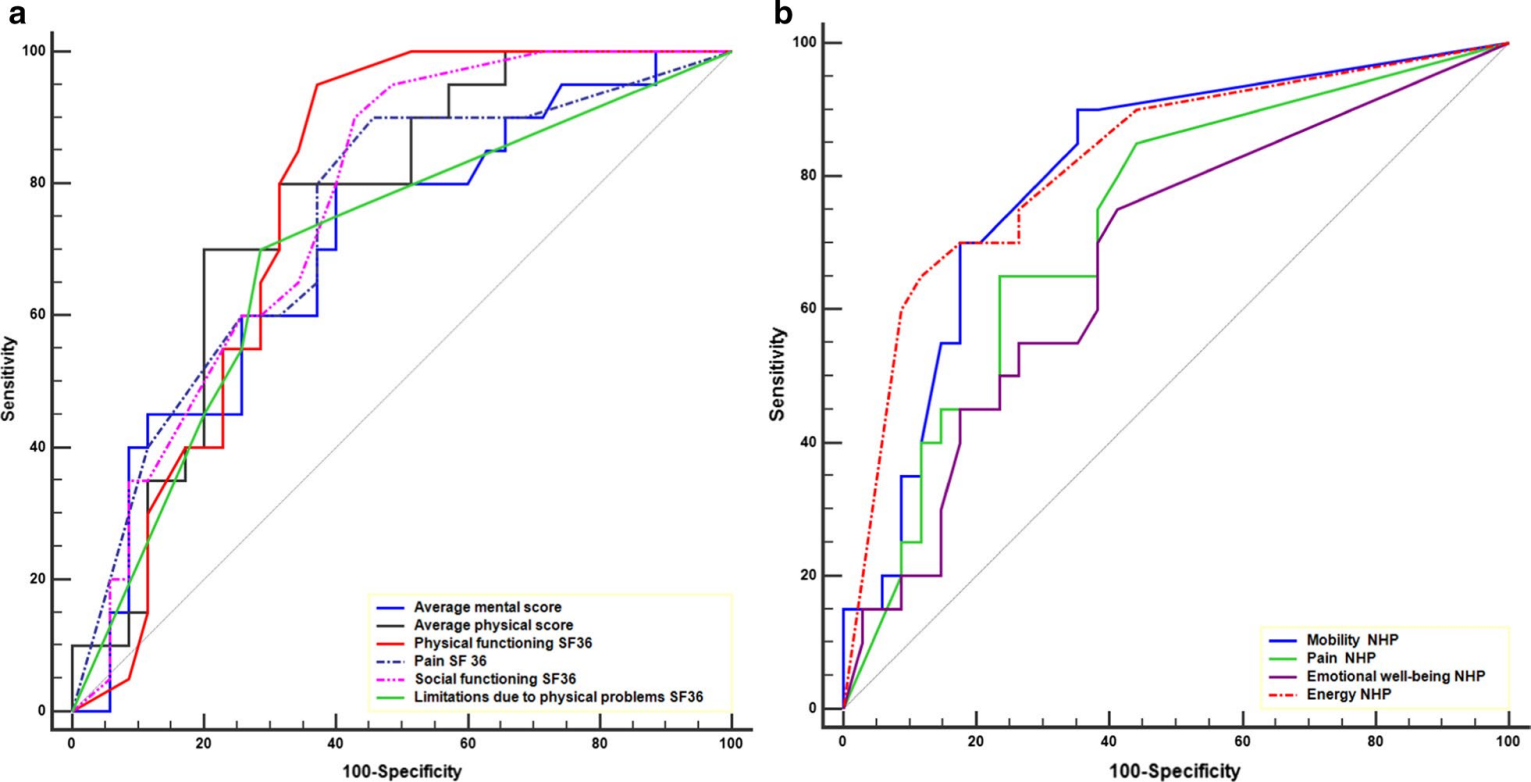
Comparison of ROC curves of statistically significant MOS SF-36 (**a**) and NHP (**b**) scores

A univariate analysis was done by completing ROC curves (Fig. 2) for every statistically different dimension of the MOS SF-36 and NHP scale in the disabled group.

The dimensions for “physical functioning” with a threshold ≤ 85 and “social functioning” with a threshold ≤ 75 items of the MOS SF-36 scores as well as the dimensions for “mobility” with a threshold > 8.5 and “energy” with a threshold > 61 of the NHP score were the most associated with disability (Table 5).

**Table 5:** Areas under the ROC curve for dimensions associated with disability

	AUC	95% CI	p	Threshold	Se	Sp	PPV	PNV
*SF36*								
Physical pain	0.74	0.61–0.85	0.0005*	≤ 66.5	90	54.29	52.9	90.5
Physical functioning	0.77	0.64–0.88	< 0.0001*	≤ 85	95	62.86	59.4	95.7
Social functioning	0.77	0.63–0.87	< 0.0001*	≤ 75	90	57.1	54.5	90.9
Role limitations due to physical problems	0.70	0.56–0.82	0.0050*	≤ 50	70	71.4	58.3	80.6
Average physical score	0.76	0.63–0.87	0.0001*	≤ 47.3	70	80	66.7	82.4
Average emotional score	0.7	0.57–0.82	0.0057*	≤ 62.2	80	60	53.3	84
*NHP*								
Mobility	0.81	0.68–0.9	< 0.0001*	> 8.51	90	65.7	60	92
Pain	0.73	0.59–0.84	0.0009*	> 29.54	65	77.1	61.9	79.4
Energy	0.82	0.69–0.91	< 0.0001*	> 61.02	65	88.2	76.5	81.1
Emotional reactions	0.68	0.54–0.80	0.0131*	> 0	75	60	51.7	80.8

AUC, 95%CI and threshold values expressed in numerical values. Se, Sp, VPP and VPN expressed in percentages. *p < 0.05. Se, sensibility; Sp, specificity; PPV, positive predictive value and NPV, negative predictive value

According to the multivariate analysis (Table 6), the physical functioning item with a threshold less than or equal to 85 was statistically predictive of disability. It was noted that the statistical model presented has an AUC (Area under the curve) of 0.87 which allows classification of 81.5% of the patients in the correct category.

**Table 6:** Multivariate analysis of predictive factors of disability based on the quality of life scores

	OR	95% CI	*p* value
Average physical score ≤ 47.3	1.7	0.26–11.13	0.5799
Energy NHP > 61.02	5.9	0.85–40.9	0.0733
Pain NHP > 29.54	0.43	0.05–3.73	0.4419
Physical functioning SF36 ≤ 85	16.5	1.43–191.32	0.0247*

NHP: Nottingham Health Program, SF36: Short Form-36, 95% Confidence Interval, *p < 0.05

AUC of the statistical model = 0.87 which allows classification of 81.5% of the patients in the correct category

In the MOS SF-36 scale, physical functioning score lower than or equal to 85 were a predictive factor of disability and consequently a factor of altered quality of life.

## Discussion

The analysis of quality of life more than five years after moderate to severe trauma showed significant quality of life impairment with a predominant impact on physical dimensions.

Most of the sample patients were male and young which corresponds with prior studies [11, 12, 14–17].

The first meaningful result of our study was the impact of trauma on daily life. As a matter of fact, almost 17% of the patients had not resumed their professional activity, while more than 20% of the patients needed professional re-training and more than 16% had to have their work place adapted to accommodate their disability. As for patients who had resumed their professional activity, only 68% were able to undertake this on a full-time basis. These results correspond with findings in prior studies.

In fact, in 1998 and 2005, Braithwaite et al. [11] and Sluys et al. [12] gathered data that demonstrated a resumption of activity in 74% and 68% of the cases respectively for severely injured patients approximately 5 years following their injury. More recently, in a study conducted in 2016 on 147 patients with multiple traumas with medical follow-up of up to five years after the initial trauma, Zwingmann et al. [17] found a loss of professional activity in 16% of the cases.

Furthermore, a disability status was acknowledged in 36.4% of the patients in the study cohort. Note that this status is assigned to patients with a decreased capacity to work and with earnings reduced by at least two thirds, or due to their health status after an accident or a non-occupational related illness. This status provides grounds for a disability allowance, and the allotted amount depends on the category of disability in which patients are classified. These results highlight the serious impact of trauma. The capacity to work was altered in a majority of the population studied, and this alteration in professional life generated a significant loss of income for patients, and represents a major economic and human cost for the society.

In addition, we assessed the quality of life of patients by combining two standardized scales: the MOS SF-36 and the NHP scales. These two scales were combined in order to include patients with brain or head injury. As a matter of fact, in the Gross et al. [15] study, the authors concluded that this association enabled them to carry out the most accurate assessment of quality of life in a population of multiple trauma patients presenting with a potential traumatic brain injury. Van Beeck and *coll.* propose to use a combination of EuroQol-5D and Health Utilities Mark Ill in all studies on injury-related disability [18]. Ardolino A and *coll* in a consensus meeting also suggest the use of European Quality of Life 5D (EQ-5D) with consideration to be given to the World Health Organisation Quality of Life survey (WHO-QoL) [19].

The MOS SF-36 and NHP scales provided an objective basis to arrive at the conclusion of significant quality of life impairment in the population studied. The somatic element appeared to be more affected than the emotional element, notably according to the NHP scale. In a study conducted in 2004, Dimopoulou et al. also found data indicating significant physical health impairment according to the NHP scale in a population of multiple trauma patients with medical follow-up of up to a year after the initial trauma (64% of the patients presented with impairment in health dimensions such as “mobility”, “energy” and “pain”) [16].

In the BRAIN ICU study [4], the authors demonstrated that approximately one third of the patients hospitalized in the ICU, regardless of their injury, presented with mild depression at 3 and 12 months after trauma, and that the somatic dimension was the primary factor underlining mood fluctuations. Finally, in the Gross study, 76% of the multiple trauma patients presented with chronic pain, two years after their injury [15].

When the results of the MOS SF-36 were compared with those found in the French reference population of the decennial study conducted by INSEE in 2002–2003, deterioration in quality of life also prevailed in the dimensions of the somatic components. These results seem to correspond with those obtained in the Sluys et al. study [12]. In fact, the authors identified lowered scores in all aspects of health studied, and also reported that 68% of the patients suffered from a physical disability, whereas only 41% of the patients presented with emotional impairments.

Therefore, these results highlight the fact that major trauma affects not only the somatic dimension, but the emotional dimension as well. Nevertheless, physical impairments seem to be the main source of disability perceived by patients.

Based on these findings, it could be beneficial to propose early emotional and physical rehabilitation for this population in order to decrease the impact on quality of life following hospitalization. Van der Schaaf et al. have shown that patients who received care in the ICU for at least two days are the target population for the introduction of rehabilitation programs; which include not only physical exercise aimed at improving walking and endurance, but also care for emotional distress and problems related to concentration and memory [20]. In a study published in 2012, Jackson et al. underscored the importance of rehabilitation programs dedicated to patients who had received care in the ICU. With physical, cognitive and functional training, patients’ future improved in only 3 months [21].

Finally, we specifically analyzed the subgroup of disabled patients. Our study showed that physical functioning scores with a threshold ≤ 85 on the MOS SF-36 scale were predictive of disability according to criteria established by the French Health Insurance Agency. To our knowledge, there are no studies that have specifically identified a predictive factor for disability based on quality of life assessment scales. However, these results have yet to be validated by the completion of multi-centric prospective studies.

There are several limitations in our study. First of all, it is a monocentric analysis. However, the study population seems similar to other studies conducted on cohorts of multiple trauma patients, (in terms of age, gender, length of hospitalization and the severity of the initial injury) [11, 12, 14, 16, 17]. Second, the participation rate in this study was lower than in other studies conducted on the outcomes for victims of severe injuries. However, a comparative analysis was done of patients who did not participate and those who did and the results indicated an absence of statistically significant discrepancies between the two populations. This indicates the representativeness of our cohort with respect to the total population contacted for the study. Finally, the history of somatic or psychiatric disorders in the population studied was not explored, which could have had an impact on the scores obtained from the quality of life assessment.

## Conclusion

Our study shows that more than 5 years after a moderate to severe trauma, there is a significant alteration in the quality of life. Disability secondary to major trauma is predicted by the presence of a physical functioning score ≤ 85 on the MOS SF-36 scale. This finding should be grounds for the introduction of specific rehabilitation programs (including physical and emotional components), implemented in intensive care units in order to limit the long-term consequences of major trauma on patients, and the resulting socio-economic impacts. There are differences in the long-term quality of life of trauma patients compared to the quality of life of the general French population, with the limitation that these outcomes have to been adjusted for the different age/sex categorization in the study sample versus the general population sample.

## Data Availability

The datasets used and/or analyzed in this study are available from the corresponding author on reasonable request.

## Abbreviations

ARDS: Acute Respiratory Distress Syndrome
AUC: Area under the curve
ICU: Intensive Care Unit
INSEE: French Statistics and Economic Studies Institute
ISS: Injury Severity Score
MOS SF-36: Medical Outcome Study Short Form-36
NHP: Nottingham Health Profile
PICS: Post-Intensive Care Syndrome
ROC: Receiver Operating Characteristics
SAPS II: New Simplified Acute Physiology Score II
